# Large variations between NICU nurses in predicting nasogastric tube insertion length in a mannequin study

**DOI:** 10.1016/j.ijnsa.2021.100055

**Published:** 2021-11-27

**Authors:** E.A. Lopriore, W.B. de Vries, L.E. van der Meeren, E. Lopriore, H.A. van Zanten

**Affiliations:** a Department of Neonatology, Division Women & Baby, University Medical Center Utrecht, Utrecht, the Netherlands; b Department of Pathology, University Medical Center Utrecht, Utrecht, the Netherland; c Department of Pathology, Leiden University Medical Center, Leiden, the Netherland; d Department of Pediatrics, Division of Neonatology, Leiden University Medical Center, Leiden, the Netherland

**Keywords:** Nurses, Nasogastric tube, Neonates, Interindividual variation, Mannequin study

## Abstract

**Aim:**

To evaluate the inter- and intraindividual variation of predicted nasogastric tube insertion lengths by nurses working in two neonatal intensive care units in the Netherlands, using a mannequin model.

**Methods:**

A total of 110 nurses (55 nurses from Center A and 55 from Center B) were asked to predict the nasogastric tube insertion length on a neonatal mannequin. We evaluated the length and prediction method used by the nurses. We also estimated the number of tubes that would have correctly been placed in the stomach of a neonate according to the seize of the mannequin.

**Results:**

The mean predicted insertion length of the nasogastric tube was 30.0 cm with an interindividual variation of 12 cm (range 24–36 cm). The mean intraindividual variation was 0.75 cm. The two centers used two different prediction methods in their local guidelines, but overall at least 6 different methods were used by the nurses. We estimated that 77% (85/110) of the tubes would have ended in the body of the mannequins stomach, while 10% (11/110) would have ended in the esophagus and 13% (14/110) would have ended against the stomach lining or in the duodenum.

**Conclusion:**

Nurses in two neonatal intensive care units used many different methods which lead to a large interindividual variation in predicted insertion lengths of the nasogastric tubes. Regular evaluations using this mannequin model could lead to more uniformity and reduce the risk of tube misplacement in neonates.

What is already known about this topic?

Different methods are currently used to predict the insertion length of nasogastric tubes in neonates.

Nasogastric tubes are often placed too deep or not deep enough, which may cause severe complications, especially in extreme preterm neonates.

What this paper adds

Despite national and local protocols, nurses use a large variety of different methods to predict nasogastric tube insertion lengths.

Even when the same method was used, large interindividual variation in insertion lengths was found.

Regular evaluations using this mannequin model could lead to more uniformity and reduce the risk of tube misplacement in neonates.

## 1 Introduction

Nearly all premature neonates in the neonatal intensive care unit depend on nasogastric tubes for nutrition, medication, gastric decompression or gastric lavage (de Boer et al., 2017; Nguyen et al., 2016). Nasogastric tubes are correctly placed when the tip passes the gastro-esophageal sphincter and is located in the body of the stomach (Backstrand et al., 2007). Predicting the accurate insertion length of the nasogastric tube is crucial, since incorrectly placed tubes can cause severe complications in already very fragile patients. Tubes that are not placed deep enough may end up in the esophagus, causing reflux, aspiration and/or vasovagal responses of apnea and bradycardia. Tubes that are placed too deep may damage the gastric wall or end up in the duodenum, causing malabsorption, diarrhea, and failure to gain weight (de Boer et al., 2017; Nguyen et al., 2016; Ellet et al., 2011). Misplacement of gastric tubes can even lead to severe complications, such as esophageal or gastric perforations, and even neonatal demise (Gasparella et al., 2011; Su et al., 2009).

In the neonatal intensive care units in the Netherlands, nasogastric tubes are routinely placed by nurses. All nurses need a certificate of competence for the insertion of a gastric tube in a neonate. The process for obtaining this certificate of competence contains an e-learning, combined with a theoretical and practical assessment by a colleague or supervisor. After obtaining this certificate of competence the nurse receives a certificate that is valid for 12 months. In the past, various methods were used to predict the insertion length for nasogastric tubes in neonates. One of the frequently used methods was the Nose – Earlobe – Xiphoid method (NEX). In this method the tube-length is predicted by measuring the distance from the tip of the nose to the earlobe to the xiphoid process. In 2014, a national guideline for neonates was introduced based on the Nose - Earlobe - Midway xiphoid and Umbilicus method (NEMU). ^1^ According to the NEMU method the tube-length is predicted by measuring the distance from the tip of the nose to the earlobe to a point midway in between the xiphoid process and the umbilicus. This new national guideline (de Boer et al., 2017) was based on a study of Ellet et al. (2011), in which the NEMU method was found superior to the NEX method. Nevertheless, still approximately 10% of the tubes were misplaced even by using the NEMU method. ^4^

Most previous studies have focused on comparing different prediction methods. However, the accuracy of the application of these prediction-methods by nurses, i.e. the inter- and intraindividual variation within the same method, have not yet been evaluated.

The aim of this study is to evaluate the inter- and intraindividual variation of predicted insertion lengths by nurses in two divisions of neonatology in the Netherlands, using a neonatal mannequin.

## 2 Methods

In January and February 2021, nurses and trainees working at two level III neonatal intensive care units in the Netherlands (Centers A and B) were approached to participate in the evaluation. Information about the specific aim of the study was not widely elaborated in order to obtain the most accurate results. To minimize the risk of inter-subject communication, the nurses were urged not to discuss the study with their colleagues. Nurses were guaranteed full confidentiality and we documented only their years of work-experience and whether they had finished their traineeship. In addition, all nurses and trainees were in possession of a certificate of competence for the insertion of a gastric tube in a neonate.

Each nurse was asked to predict the nasogastric tube insertion length three times on a 57 cm mannequin used to practice neonatal reanimations (Laerdal ALS Baby Trainer). Measurements were performed using a nasogastric tube (VYGON Nutrifit 05Fr L125cm PUR (REF 1362.057)), routinely used at the neonatal intensive care unit. For each nurse the three predicted insertion lengths and the difference between the highest and lowest measurement were documented to assess the intraindividual variation. The mean value of the three predicted lengths and the landmarks used for these predictions were documented to assess the interindividual variation. Additionally, we recorded whether the tension with which the tubes were held was loose, average or tight. This was estimated through observation, not through specific measurement.

To examine the potential clinical impact of the insertion length range, we estimated the number of tubes that would have been correctly placed in the stomach of the mannequin according to the predicted insertion lengths. The mannequin used was 57 cm of height, which is the average height of a 1 month-old neonate (Fenton and Kim, 2013). The height of a stomach of an infant that age would be approximately 6 cm (Cetin et al., 2006). We calculated the number and percentage of misplaced tubes, assuming that the mean predicted insertion length would place the tube in the middle of the stomach. With that assumption, insertion lengths >3 cm below the mean would hypothetically place tubes not deep enough, while insertion lengths >3 cm above the mean would place tubes to deep.

Outcome parameters were described as either absolute numbers and percentages for categorical variables, or mean, range and standard deviation (SD) for continuous and normally distributed variables. Following a non-significant Levene's test for variances, a one way ANOVA test was used to compare the insertion lengths for the most frequently used methods and a Bonferroni test was used to compare the methods pairwise. A statistical significant difference was defined as *p* < .05. Statistical analyses were carried out using SPSS version 27 (IBM, Armonk, NY, USA).

This quality study was performed on a mannequin, therefore ethical approval was not required according to our institutional guidelines. The participating nurses gave consent to record and use the data.

## 3 Results

A total of 110 nurses participated in the evaluation, 55 from Center A and 55 from Center B. In Center A and Center B, respectively 69 and 85 nurses were actively working during the study period, yielding a participation percentage of 80% (55/69) in Center A and 65% (55/85) in Center B. The average years of work-experience was 13.6 (SD 11.7) and 77% (85/110) were specialized nurses while 23% (25/110) were trainees.

**Fig. 1:**
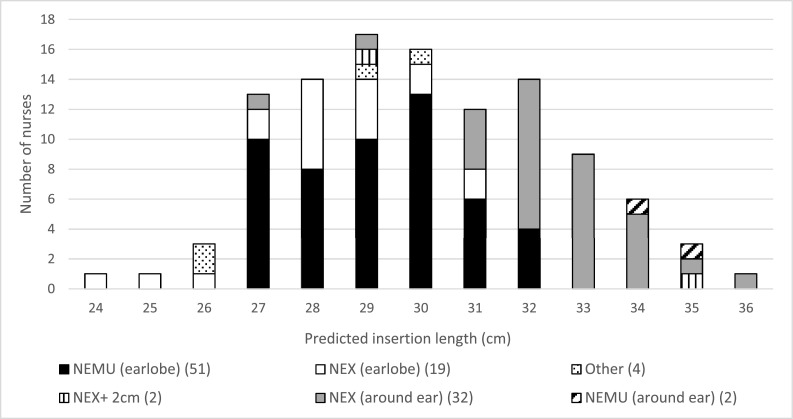
Variation of predicted insertion lengths by the nurses NEMU (= Nose – Earlobe - Midway xiphoid and Umbilicus) NEX (= Nose – Earlobe - Xiphoid).

Overall, the mean predicted length was 30.0 cm (SD 2.45 cm), with an interindividual variation of 12 cm, ranging from a minimum of 24 cm to a maximum of 36 cm (see Table 1). The variation in predicted length was normally distributed and shown in Fig. 1. The mean intraindividual variation was 0.75 cm (SD 0.77 cm).

**Table 1:** Predicted insertion lengths and ranges according to different methods used by the nurses. NEMU (= Nose – Earlobe - Midway xiphoid and Umbilicus) NEX (= Nose – Earlobe - Xiphoid).

Method used	Number of nurses, n (%)	Predicted Length, mean (SD), cm	Range (minimum and maximum), cm
1: NEMU earlobe*	51 (46)	29.2 (1.54)	5 (27–32)
2: NEMU around ear	2 (2)	34.7 (0.94)	1 (34–35)
3: NEX around ear**	32 (29)	32.4 (1.57)	9 (27–36)
4: NEX earlobe	19 (17)	28.0 (1.81)	7 (24–31)
5: NEX + 2 cm	2 (2)	31.9 (4.36)	6 (29–35)
6: Other	4 (4)	27.8 (2.03)	4 (26–30)
Total	110 (100)	30.0 (2.45)	12 (24–36)

*NEMU earlobe: current national guideline used in center A.

**NEX around ear: local guideline used in center B.

### 3.1 Variation in measuring methods

Of the 110 nurses, only 51 (46%) used the current national guideline (the NEMU method) and 2 nurses (2%) used the NEMU method but incorrectly measured around the ear. A total of 51 (46%) nurses used the NEX method, of which 37% (19/51) of nurses used it from nose to earlobe and to xyphoid, while 63% (32/51) used the NEX method but measured around the ear instead of to the earlobe. Two nurses (2%) used the NEX method, one around the ear and one along the earlobe, and then added 2 cm (a method called: NEX + 2 cm). Four nurses (4%) used a different measuring order that started from the ear, continued to the nose and ended at the xiphoid, umbilicus or a point midway the xiphoid and umbilicus.

Most nurses in Center A (87%, 48/55) followed the current national guideline (NEMU method), compared to 6% (3/55) of nurses from Center B. With hindsight, we found that Center B did not use the national guideline but a local guideline based on the adapted version of the NEX method, which explains part of the interindividual variation. In Center B, the local protocol makes a distinction between the method used for nasogastric tubes (placed through the nose, as in this mannequin study) and orogastric tubes (placed through the mouth). For nasogastric tubes, the local protocol in Center B dictates the use of the NEX method around the ear, whereas for orogastric tubes, the local protocol suggests a different measuring method, from mouth to earlobe to xiphoid instead of around the ear. In Center B, 58% (32/55) of nurses used the local protocol (NEX around the ear) whereas 33% (18/55) used the NEX method to the earlobe, which resembles their local protocol for orogastric tubes instead of nasogastric tubes. The national protocol (the one also used in Center A) uses the same predicting methods for oro- or nasogastric tubes, without distinction between the two types.

In addition to these larger method variations, we also recorded smaller variations in the measuring methods. Not all nurses used the tip of the nose as a landmark (24%, 26/110), but some nurses (10%, 11/110) measured from the lateral point of the nose wing and 63% (69/110) measured from the nostril. Also 15% (16/110) of nurses did not measure in a direct line from ear to stomach, but measured along the chin. The majority of nurses used an average tension on the nasogastric tube when measuring the predicted length, while 35% (38/110) of the nurses pulled firmly on the gastric tube and 16% (17/110) held the tube loosely.

### 3.2 Variation in prediction length according to various methods

The most frequently used methods were the NEMU method along the earlobe and the two NEX methods. The mean of the insertion lengths predicted with the NEMU method was 29.2 cm (range 5 cm from 27 to 32). The mean insertion length predicted with the NEX method around the ear was 32.4 cm (range 9 cm from 27 to 36) and with the NEX method along the earlobe 28.0 cm (range 7 cm from 24 to 31).

**Fig. 2:**
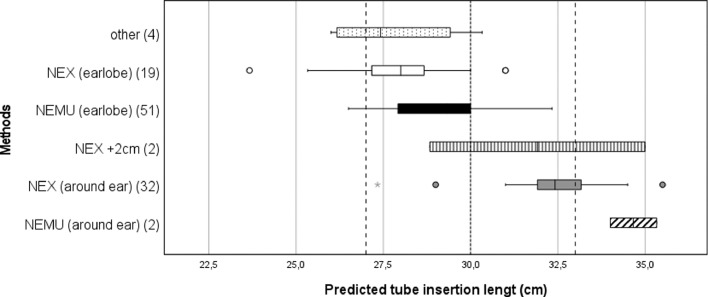
Variation of measured insertion lengths according to different methods. (*x* = 27: assumed esophagogastric junction; *x* = 30: mean predicted insertion length; *x* = 33: assumed distal margin of the body of the stomach) NEMU (= Nose – Earlobe - Midway xiphoid and Umbilicus) NEX (= Nose – Earlobe - Xiphoid).

The one way ANOVA test was executed after finding no significant difference between the variances of these methods (Levene's test *p* = 65). The insertion lengths were significantly different (ANOVA *p* = 00), and the methods were individually significantly different in the paired comparison as well (Bonferroni NEMU – NEX around ear *p* = 00; NEMU – NEX along earlobe *p* = 03; NEX around ear- NEX along earlobe *p* = 00), showing that the insertion lengths from shortest to longest were respectively the NEX method to earlobe, the NEMU method and the NEX method around the ear. The variation in prediction length according to the method used is shown in Fig. 2.

### 3.3 Hypothetical correct position in stomach

To examine the potential clinical impact of the insertion length range, the number of correctly placed tubes was estimated. Assuming that the height of the mannequin's stomach was approximately 6 cm and the mean of all predicted insertion lengths 30 cm would have ended in the middle of the stomach, all tubes inserted ≤27 cm would have ended in the esophagus (10% (11/110)) and all tubes inserted ≥33 cm would have ended against the stomach lining or in the duodenum (13% (14/110)), whereas the tip of the nasogastric tube would have correctly ended in the body of the stomach in 77% (85/110).

## 4 Discussion

In this mannequin study we found an acceptable mean intraindividual variation of 0.75 cm but a large interindividual range of 12 cm in predicted insertion lengths of nasogastric tubes. Not only did we discover major differences in the guidelines used in the two centers, but we also found differences in the application of these guidelines by the nurses. These differences range from holding the nasogastric tube with a different tension and interpreting landmarks differently (e.g. the nose wing instead of the tip of the nose), to using different landmarks than defined in their local protocols or even different measuring orders. This resulted in the use of at least 6 different measuring methods. Even when the same method was used, we still found an interindividual range of 5 cm with the NEMU method, and 7 and 9 cm with the NEX method respectively around the ear and to the earlobe.

Considering the average 6 cm height of an infant's stomach, such large ranges could potentially lead to severe complications due to nasogastric tube misplacement. When we estimated the amount of tube-lengths measured on the mannequin that would have been placed correctly, we found that only 77% of the tubes would have ended correctly in the body of the stomach. These results found in our *in vitro* study are similar to the misplacement rates found by previous studies comparing different prediction methods *in vivo*, as Ellet et al. (2011) found that the NEX method (along the earlobe) placed 60.6% of the tubes correctly, while the NEMU method placed 90.9% correctly.

Despite the inaccuracy of these prediction-methods being known for many years and the numerous researches conducted on finding better prediction methods, the variation between nurses has not been evaluated *in vitro* before. Evidently, research comparing predicting methods *in vivo* on real infants should be followed closely and the best evidence proved prediction method should be implemented nationally. However, we also suggest that tube misplacement could be decreased by studying the variation of the method *in vitro* with this mannequin study. Furthermore, alterative prediction formulas basing the insertion length on age and height of a neonate could also be less prone to variation and different adaptations (Nguyen et al., 2016; Ellet et al., 2011). The accuracy and intraindividual variations of these alternative prediction formulas should be evaluated as well.

One limitation of this study was the participation of only two centers. Including more neonatal intensive care units in the Netherlands would be valuable to obtain greater national results about the variation between nurses, and to evaluate the uniform implementation of the national guideline. Nevertheless, the variation revealed by this mannequin study calls for instant simple improvements. To date, only one or two sentences in the gastric tube protocol are contributed to the crucial process of predicting the correct insertion length. By describing the prediction methods in more detail in the protocol, less parts of the method would be open to interpretation or cause variation. This could also be added to the procedure for obtaining a certificate of competence for gastric tube insertion. Furthermore, this mannequin study provides a great form to evaluate the variation between used predicting methods by nurses on the neonatal units every few years. By increasing the awareness and transparency in such manner, the interindividual variation could be decreased and uniform gastric tube procedures for these smallest patients could be ensured. In conclusion, nurses used many different methods which lead to a large interindividual variation in predicted insertion lengths of the nasogastric tubes. A detailed instruction (including illustrations) in the current guideline, combined with regular evaluations using a mannequin model could lead to more uniformity and reduce the risk of tube misplacement in neonates.

## Declaration of Competing Interest

None.
